# Characterizing Stage‐Specific Cellular Dynamics and Microenvironmental Remodeling in Lung Adenocarcinoma by Single‐Cell RNA Sequencing

**DOI:** 10.1002/advs.202510847

**Published:** 2025-11-28

**Authors:** Bomiao Qing, Xiaolan Li, Xiang He, Junyi Wang, Yi Yang, Manling Jiang, Bingbing Yan, Lei Zhang, Anying Xiong, Qin Ran, Guoping Li

**Affiliations:** ^1^ Laboratory of Allergy and Precision Medicine Chengdu Institute of Respiratory Health Affiliated Hospital of Southwest Jiaotong University The Third People's Hospital of Chengdu Chengdu 610031 China; ^2^ Laboratory of Allergy and Precision Medicine Chengdu Institute of Respiratory Health The Third People's Hospital of Chengdu (Affiliated Hospital of Southwest Jiaotong University) College of Medicine Southwest Jiaotong University Chengdu 610031 China; ^3^ Department of Pulmonary and Critical Care Medicine Chengdu Third People's Hospital Branch of National Clinical Research Center for Respiratory Disease Affiliated Hospital of Chongqing Medical University Chengdu 610031 China; ^4^ Department of Thoracic Surgery Affiliated Hospital of Southwest Jiaotong University The Third People's Hospital of Chengdu Chengdu 610031 China; ^5^ Clinical Medicine Department North Sichuan Medical College Nanchong 637000 China

**Keywords:** hypoxia, LUAD, single‐cell RNA sequencing, TME

## Abstract

Lung adenocarcinoma (LUAD) progression involves dynamic remodeling of the tumor microenvironment (TME). However, the stage‐specific dynamics of immune and stromal cell remodeling throughout LUAD progression remain incompletely understood. Here, the study systematically profiles the cellular composition and transcriptional states across multiple LUAD stages, integrating early‐stage patient specimens with publicly available datasets encompassing advanced‐stage disease. The analysis reveals a marked stage‐dependent shift from a proliferative and immune‐activated microenvironment in early LUAD to a hypoxia‐enriched and immunosuppressive landscape in advanced disease. A distinct hypoxia‐adapted epithelial tumor cell subpopulation (C5), exhibiting transcriptional features of metastasis, invasion, and hypoxia, and poor prognosis, is identified. Advanced LUAD featured immunosuppressive LGMN⁺ macrophages and STAT1‐driven exhausted CD8⁺ T cells. FKBP11⁺ plasma B cells exhibited exhaustion‐linked metabolic changes. POSTN⁺ CAFs emerged as central mediators of extracellular matrix (ECM) remodeling and immune exclusion. Collectively, the findings reveal a model of hypoxia‐driven functional convergence, in which distinct TME components co‐evolve toward phenotypes that collectively promote immune evasion, matrix remodeling, and tumor progression. These findings may provide insights into stage‐specific cellular dynamics and highlight promising therapeutic targets for precision immunotherapy strategies.

## Graphical abstract

This study integrated early‐stage patient specimens with publicly available datasets encompassing non‐malignant lung tissues and advanced‐stage LUAD through single‐cell sequencing, revealing a dynamically remodeled tumor microenvironment characterized by a hypoxia‐adapted malignant subset (C5), immunosuppressive LGMN^+^ macrophages, STAT1‐driven exhausted CD8^+^ T cells, dysfunctional FKBP11^+^ plasma cells, and pro‐metastatic POSTN^+^ CAFs, collectively highlighting coordinated hypoxia‐driven progression and immune evasion.

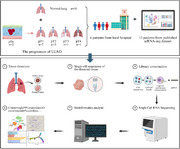

## 1 Introduction

Lung cancer is one of the most burdensome cancers for global healthcare systems, imposing significant challenges due to its high incidence, mortality rate, and the complexity of treatment.^[^ ^1^ ^]^ Lung adenocarcinoma (LUAD) is the most common subtype of lung cancer, accounting for approximately 40–60% of newly diagnosed lung cancer cases, and is often associated with specific genetic mutations that influence treatment strategies.^[^ ^2^, ^3^ ^]^ The development of LUAD follows a multi‐step pathological process, typically progressing from atypical adenomatous hyperplasia (AAH) to adenocarcinoma in situ (AIS), then to minimally invasive adenocarcinoma (MIA), and ultimately culminating in invasive adenocarcinoma (IAC).^[^ ^4^ ^]^ This sequential progression is driven by profound cellular and molecular remodeling within the tumor microenvironment (TME), which orchestrates immune interactions and facilitates tumor initiation and invasion.^[^ ^5^ ^]^

The LUAD TME constitutes a highly dynamic ecosystem comprising malignant cells, various immune cells such as T cells, B cells, macrophages, and NK cells, as well as the extracellular matrix and biomolecules.^[^ ^6^ ^]^ During tumor progression, the composition, phenotype, and functional state of these cellular populations undergo continuous remodeling, reflecting an evolving balance between immune surveillance and tumor‐promoting signals.^[^ ^7^ ^]^ Tumor cells facilitate immune evasion by suppressing immune cell function, promoting immune escape, and modulating the immune response.^[^ ^8^ ^]^ Consequently, immune cells within the TME may undergo progressive dysfunction and exhaustion, leading to attenuated anti‐tumor activity and diminished responses to immunotherapies such as immune checkpoint blockade (ICB). Therefore, a comprehensive delineation of these dynamic immune–stromal interactions at single‐cell resolution is essential for identifying novel therapeutic targets and optimizing immunotherapy strategies.

Despite growing insights into the TME, particularly in advanced LUAD stages, the dynamic evolution of the TME from premalignant lesions to invasive carcinoma remains insufficiently characterized. Recently, single‐cell RNA‐sequencing (scRNA‐seq) has emerged as a powerful tool to profile the TME of LUAD.^[^ ^7^, ^9^ ^]^ This technology enables the identification of the transcriptional characteristics of different cell populations during tumor development and uncovers the interactions between cells, providing insights into how the TME regulates tumor growth and metastasis through cytokines and direct cell–cell interactions.^[^ ^10^, ^11^ ^]^

Here, we employed scRNA‐seq to systematically characterize the TME across multiple histological stages of LUAD, including non‐malignant lung tissue, early‐stage LUAD, and advanced LUAD. We constructed a high‐resolution single‐cell transcriptomic atlas that delineates the dynamic remodeling of the cellular composition, transcriptional states, and functional phenotypes during tumor progression, and further comprehensively analyzed the molecular features of malignant epithelial cells and the lineage diversity of immune and stromal compartments within the evolving TME. Our study provides mechanistic insights into LUAD progression and immune remodeling, offering a valuable resource for biomarker discovery and the development of stage‐specific precision immunotherapies.

## 2 Results

### 2.1 Single‐Cell RNA‐Seq Analysis Reveals the Stage‐Specific Immunogenomic Landscape of Lung Adenocarcinomas

**Figure 1:**
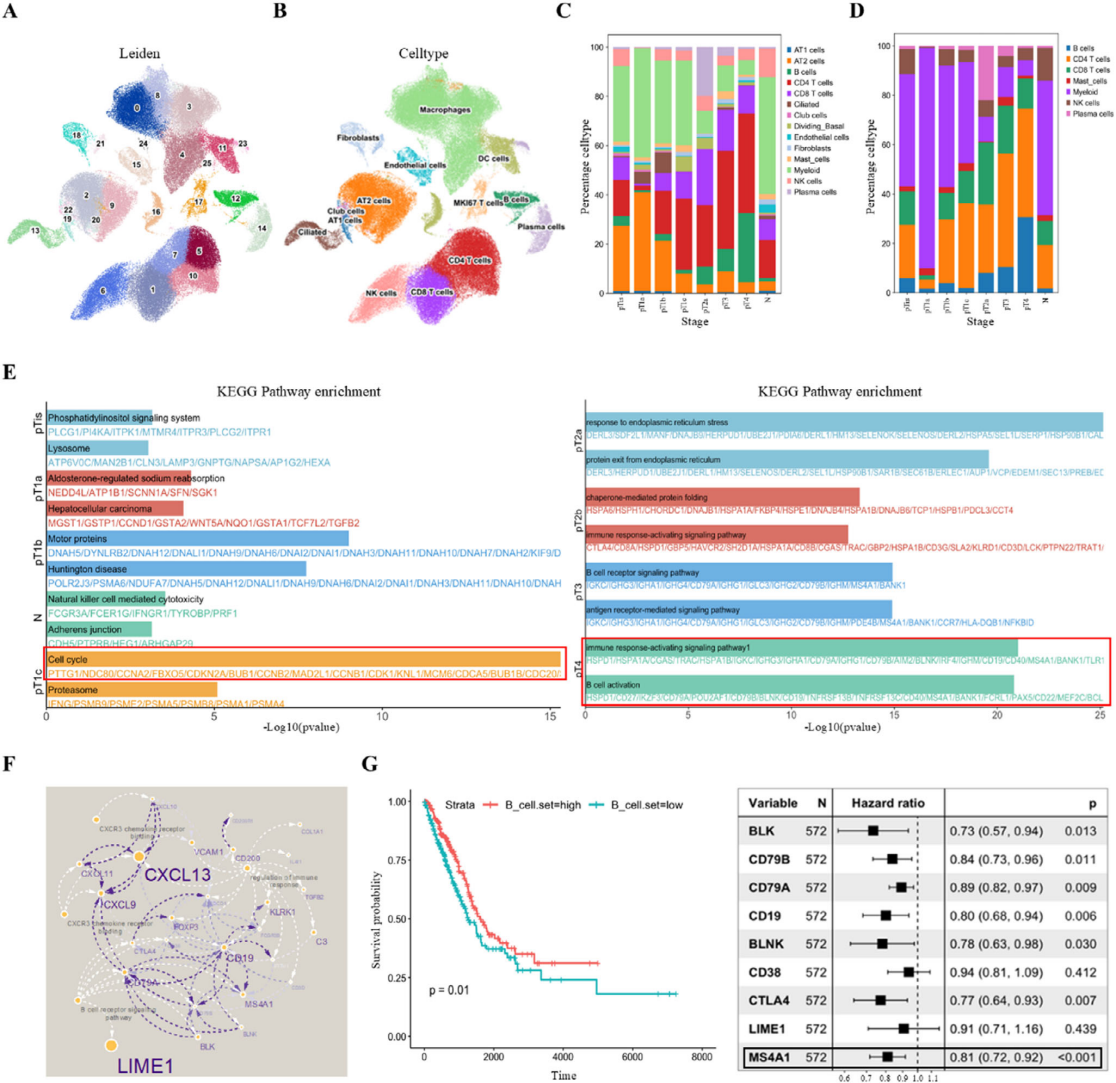
Single‐cell transcriptomic landscape of lung tissues across LUAD progression. A, B) UMAP plots showing unsupervised clustering using the Leiden algorithm (A) and cell type annotation based on canonical markers (B). C, D) Relative proportions of major cell types (C) and immune cell subclusters (D) across LUAD progression stages. E) KEGG pathway enrichment analysis of DEGs across LUAD progression stages. F) PPI networks of LUAD‐upregulated genes associated with enriched GO terms. G) KaplanMeier survival analysis of TCGA LUAD patients (n = 572) with stratified by high versus low BCR pathway gene set expression (left). P‐value was calculated using the two‐sided log‐rank test. Forest plot of univariate Cox regression for BCR signalingassociated genes. HRs and 95% CIs are shown (right).

To investigate the stage‐specific single‐cell transcriptional landscape of LUAD, we performed scRNA‐seq on surgically resected lung tumor specimens from six patients with CT‐confirmed pulmonary nodules measuring less than 2 cm in diameter. These data were subsequently integrated with scRNA‐seq profiles from 12 additional LUAD patients (ten normal and ten tumor tissue samples) obtained from the publicly available dataset for comprehensive analysis (Table S1, Supporting Information).^[^ ^41^ ^]^ As shown in the Uniform Manifold Approximation and Projection (UMAP) plots, cells from different patients were well mixed, suggesting effective removal of batch effects and successful data integration (Figure S1A, Supporting Information). In contrast, stage‐based coloring revealed distinct spatial segregation of cells, suggesting potential stage‐associated transcriptional divergence (Figure S1B, Supporting Information). In total, 123725 cells derived from 18 patient samples were included for downstream analyses. Unbiased dimensionality reduction and clustering identified twenty‐six cell clusters (cluster 0–25) (**Figure** **1**A). Based on the expression of canonical marker genes, these clusters were annotated as dendritic cells, macrophages, mast cells, B cells, plasma cells, dividing cells, natural killer (NK) cells, CD4^+^ T cells, CD8^+^ T cells, ciliated, alveolar type I (AT1) cells, alveolar type II (AT2) cells, club cells, endothelial cells, and fibroblasts (Figure 1B). Compared with early‐stage LUAD (pTis–pT1c), advanced disease stages (pT2a–pT4) exhibited higher proportion of immune cell populations, particularly B cells, CD4⁺ T cells, and CD8⁺ T cells (Figure 1C,D). Kyoto Encyclopedia of Genes and Genomes (KEGG) pathway enrichment analysis revealed that upregulated genes in pT1c patients were significantly enriched in cell cycle regulation, whereas those in pT2b–pT4 patients showed prominent enrichment in immune response‐activating signaling pathways and B cell activation, indicating progressive activation of adaptive immunity in advanced disease stages (pT2b–pT4) (Figure 1E). Protein–protein interaction (PPI) network analysis further identified CXCL13, CXCL9, and CXCL11 as key nodes of CXCR3‐mediated chemotaxis modules, while CD79A, LIME1, and MS4A1 formed a functional hub in B cell receptor (BCR) signaling networks (Figure 1F). To evaluate the prognostic value of BCR pathway‐associated genes, we performed univariate Cox proportional hazards regression analysis in a cohort of 572 LUAD patients, revealing that patients with elevated expression of BCR pathway genes exhibited superior overall survival (p = 0.01) (Figure 1G). Among them, MS4A1, encodes the transmembrane protein CD20, which is highly expressed on mature B cells and plays a modulatory role in BCR signaling.^[^ ^12^ ^]^ Notably, MS4A1 emerged as an independent favorable prognostic biomarker in LUAD (HR = 0.81, 95% CI 0.72–0.92; Cox p = 3.7 × 10^−^⁴, Figure 1G). These results indicate that LUAD undergoes stage‐specific immunogenomic evolution, wherein early tumorigenesis was associated with cell cycle dysregulation, while progressive disease was characterized by enhanced adaptive immunity response.

### 2.2 Single‐Cell RNA‐Seq Analysis Reveals the Stage‐Specific Heterogeneity of Tumor Epithelial Cells in Lung Adenocarcinomas

**Figure 2:**
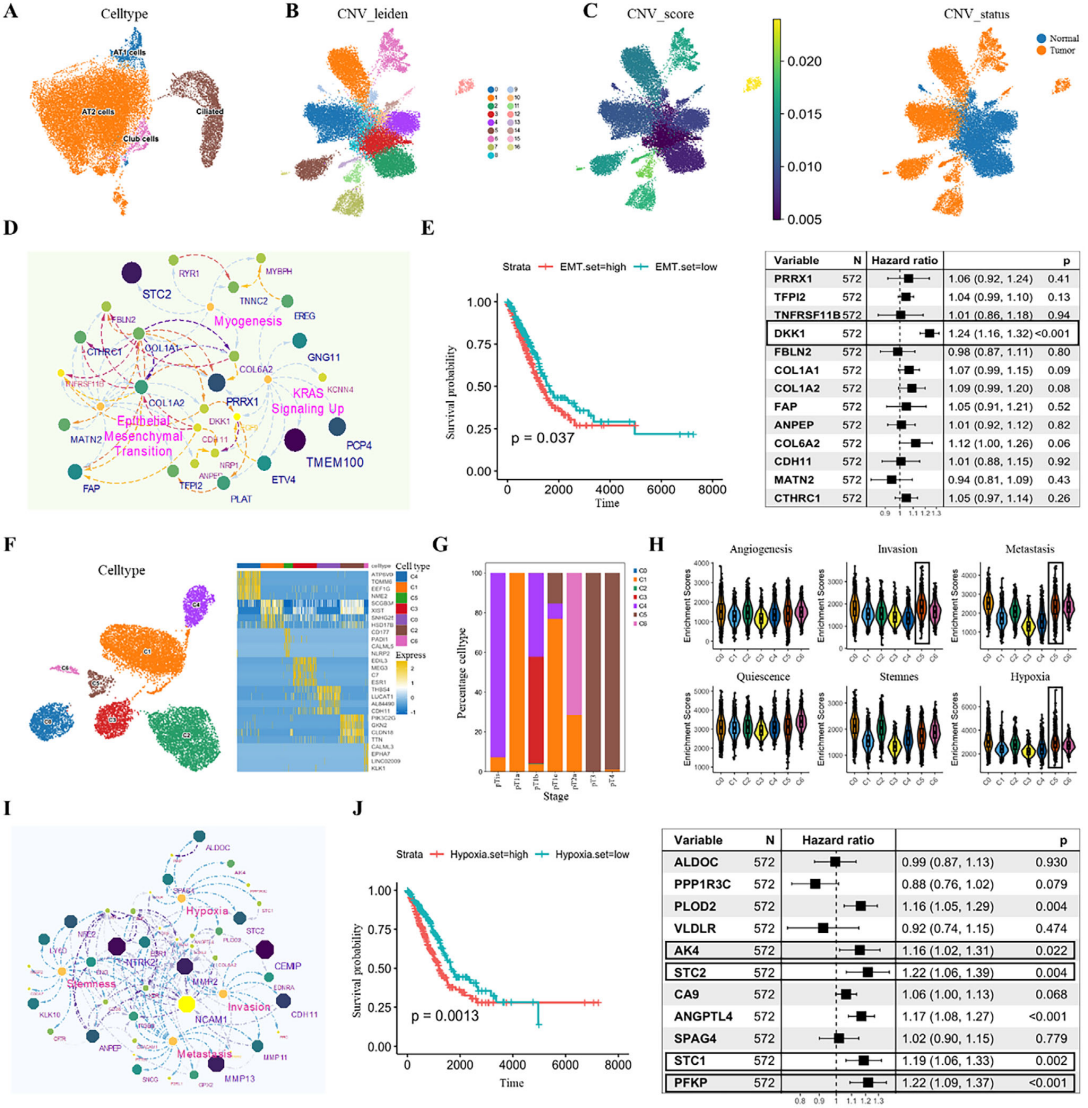
Single‐cell analysis identifies stage‐specific epithelial tumor cell states and functional programs in LUAD. A) UMAP plots showing the distribution of four epithelial cell subclusters, including ciliated cells, club cells, AT1 cells, and AT2 cells. B,C) Identification of malignant epithelial cells based on CNV. (B) CNV‐based Leiden clustering of epithelial cells. (C) CNV score (left) and predicted CNV status (right) distinguishing tumor cells (orange) from normal epithelial cells (blue). D) The PPI networks of genes upregulated in tumor cells, enriched in GO terms. Network nodes representing proteins enriched in GO terms. The Node size and color intensity represent the fold change in gene expression levels. E) Kaplan–Meier survival analysis of TCGA LUAD patients (n = 572) stratified by high versus low EMT gene set expression (left). Forest plot showing univariate Cox regression results for selected EMT‐related genes (right). HRs and 95% CIs are shown. F) UMAP plot illustrating subsets of epithelial tumor cells derived from 16 LUAD samples (left), and the heat map showing marker genes in seven subclusters (right). G) Relative percentage of seven subclusters of tumor cells across LUAD progression stages. H) The violin plots comparing the expression levels of representative genes from six classical tumor‐related functional gene sets across each tumor cell subpopulation. Each black dot represents an individual patient. I) The PPI networks of significantly enriched GO term of genes upregulated in C5 tumor cell subpopulations. The graph size and color depth representing the fold change in gene expression levels. J) Kaplan–Meier survival analysis comparing of TCGA LUAD patients (n = 572) with high and low hypoxia gene set expression (left). *P* value was calculated using the two‐sided log‐rank test. Forest plot of univariate Cox regression for hypoxia‐related genes. HRs and 95% CIs are shown (right).

Tumor cells in LUAD originate from epithelial cells and share numerous genetic features with normal epithelial populations.^[^ ^13^ ^]^ In the normal lung epithelium, four major subsets were identified, including AT1 cells, AT2 cells, club cells, and ciliated cells (**Figure** **2**A). To distinguish tumor cells from normal epithelial cells, we performed copy number variations (CNV) analysis and identified a total of 12566 tumor cells in epithelial cell population (Figure 2B,C). Compared with normal epithelial populations, Gene Ontology (GO) functional analysis revealed that genes upregulated in tumor cells were significantly enriched in epithelial‐mesenchymal transition (EMT), KRAS signaling up, and myogenesis (Figure 2D). Clinically, patients with elevated expression of EMT genes exhibited significantly reduced overall survival (p = 0.037). Strikingly, DKK1, a well‐characterized antagonist of the Wnt/β‐catenin signaling pathway and a key regulator of EMT,^[^ ^14^ ^]^ was identified as an independent poor prognostic biomarker in tumor cells (HR = 1.24, 95% CI 1.16–1.32; *p* < 0.001, Figure 2E).

To further characterize the tumor cell heterogeneity in LUAD, we performed unsupervised subclustering of tumor cells, which delineated seven transcriptionally distinct tumor cell subsets, designated as clusters C0–C6 (Figure 2F). Stage‐specific analysis indicated that the C5 subset was prominently enriched in advanced‐stage tumors (pT3–pT4) compared to early‐stage tumors (pTis–pT1b) (Figure 2G). Gene set enrichment scores were calculated based on cancer‐associated gene sets with the Escape R package (v1.8.0). C5 tumor cell subsets showed elevated activity in metastasis, invasion, and hypoxia‐related pathways (Figure 2H). PPI network analysis identified MMP11, MMP13, and NCAM1 as key nodes of metastasis and invasion modules, while STC2, ALDOC, and SPAG4 formed a functional hub in hypoxia (Figure 2I). Clinically, patients with high expression levels of hypoxia‐associated gene sets exhibited poor overall survival in TCGA LUAD cohort (*p* = 0.0013). Notably, elevated expression of the hypoxia‐associated genes AK4, STC2, STC1, and PFKP were identified as an independent poor prognostic biomarker in LUAD (Figure 2J). These findings suggest that advanced LUAD stages (pT3–pT4) were characterized by enhanced metastatic potential, increased invasiveness, and adaptive responses to hypoxic stress.

### 2.3 Single‐Cell RNA‐Seq Analysis Reveals Disease‐Specific Macrophage Subsets in Lung Adenocarcinomas

**Figure 3:**
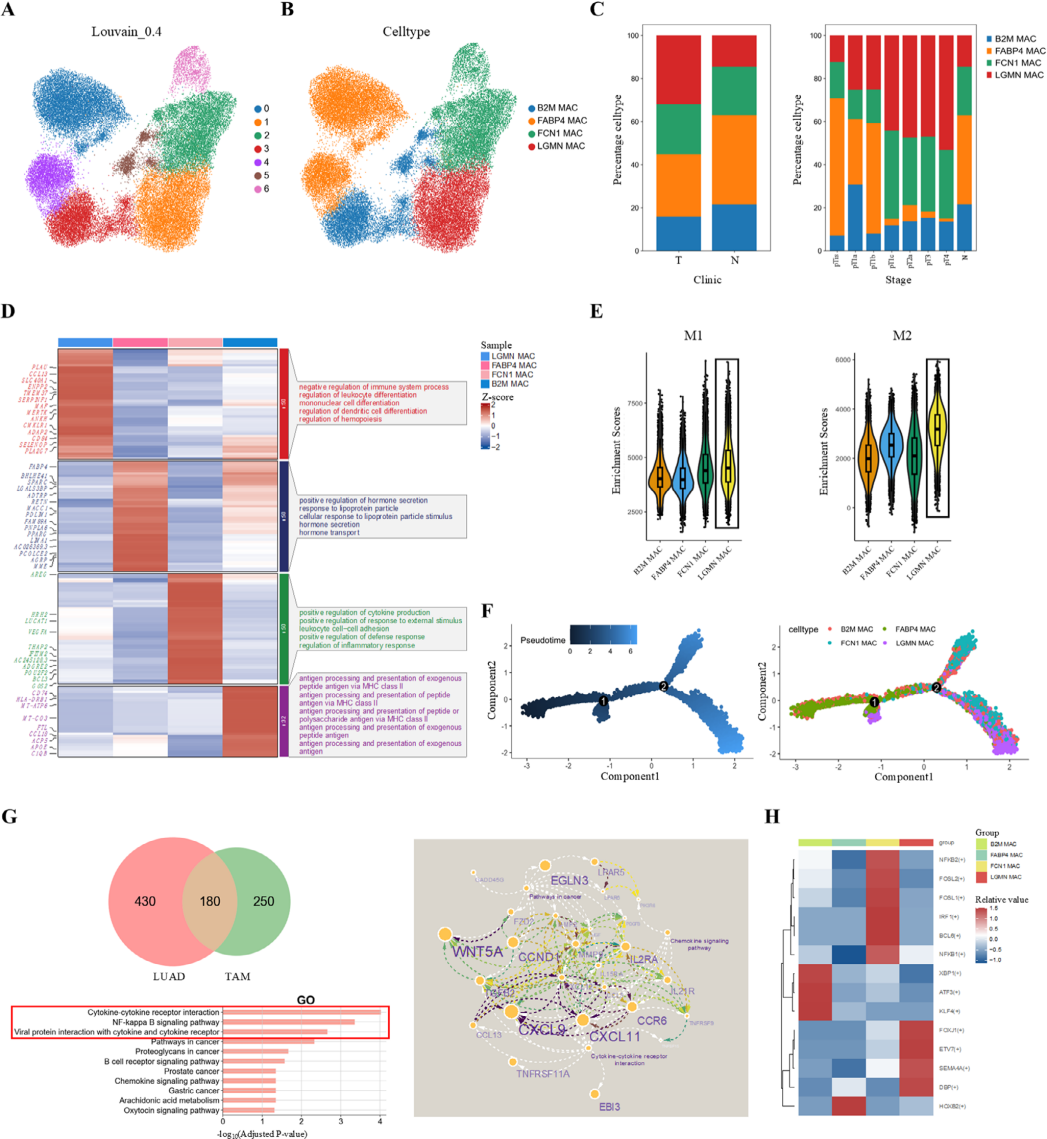
Single‐cell analysis reveals the phenotypic and functional heterogeneity of tumor‐associated macrophages in LUAD. A,B) UMAP plots showing the TAM clustering (A) and annotation of cell types based on canonical marker gene expression (B), identifying four TAM subsets: B2M⁺ MAC, FABP4⁺ MAC, FCN1⁺ MAC, and LGMN⁺ MAC. C) Relative proportions of the four TAM subsets across samples, stratified by tissue origin (left) and pathological T stage (right). D) The heatmap displaying GO terms enriched among upregulated genes in each of the four TAM subsets. E) The violin plots showing enrichment scores of M1‐ and M2‐associated gene signatures. Each black dot represents an individual patient. F) Pseudotime trajectory analysis constructed using Monocle 2 reveals the differentiation landscape of TAM subsets. Cells are colored by pseudotime values (left) and annotated TAM subpopulations (right). G) Venn diagram (upper left) showing overlapping genes between LUAD‐specific DEGs and LGMN⁺ TAM DEGs. GO enrichment analysis (lower left) and PPI network (right) of 180 overlapping genes identified in LUAD and LGMN⁺ TAMs. H) Heatmap showing expression levels of representative transcription factors predicted by SCENIC analysis to regulate the four TAM subsets.

Tumor‐associated macrophages (TAMs) constitute a major immune cell population within the TME.^[^ ^15^ ^]^ To delineate the functional heterogeneity of macrophage subsets in LUAD, macrophages were clustered into seven distinct subsets (clusters 0–6) via unsupervised dimensionality reduction (**Figure** **3**A). Four functionally specialized TAMs subsets—B2M⁺ MAC, FABP4⁺ MAC, FCN1⁺ MAC, and LGMN⁺ MAC were identified based on canonical marker gene expression (Figure 3B). Compared to normal lung tissues, tumor samples exhibited an increased proportion of LGMN⁺ MAC subclusters. Notably, the proportions of LGMN⁺ MAC and FCN1⁺ MAC were significantly elevated in advanced disease stages (pT1c–pT4) (Figure 3C). GO analysis revealed that upregulated genes in LGMN⁺ MAC were enriched in processes such as negative regulation of immune responses, monocyte and dendritic cell differentiation, and immune homeostasis maintenance (Figure 3D). Phenotypic evaluation using M1/M2 polarization marker gene sets suggested that LGMN⁺ MAC displayed transcriptional features consistent with an M2‐like phenotype (Figure 3E). Pseudotime trajectory analysis positioned LGMN⁺ MAC toward a terminal‐like state within the macrophage differentiation trajectory, suggesting potential late‐stage differentiation during LUAD progression (Figure 3F). To further explore the functional relevance of this subset, we intersected 610 LUAD‐specific differentially expressed genes (DEGs) with 430 DEGs from LGMN⁺ MAC, identifying 180 overlapping genes. These genes were enriched in pathways related to cytokine‐cytokine receptor interactions, NF‐κB signaling, and viral protein‐cytokine pathways (Figure 3G). PPI network analysis identified WNT5A, EGLN3, and CCND1 as central nodes within cancer‐related pathways, while CXCL9, CXCL11, and CCR6 were enriched in cytokine signaling (Figure 3G). SCENIC analysis further predicted FOXJ1, ETV7, DBP, and SEMA4A as potential transcription factors regulating LGMN⁺ MAC (Figure 3H). Our findings reveal that LGMN⁺ MAC was associated with LUAD progression, exhibited immunosuppressive and M2‐like features, and potentially contributing to tumor‐associated immune regulation through distinct transcription factors and signaling pathways.

### 2.4 Single‐Cell Transcriptional Profiling Delineates T Lymphocytes Heterogeneity and Functional Dynamics in LUAD Pathogenesis

**Figure 4:**
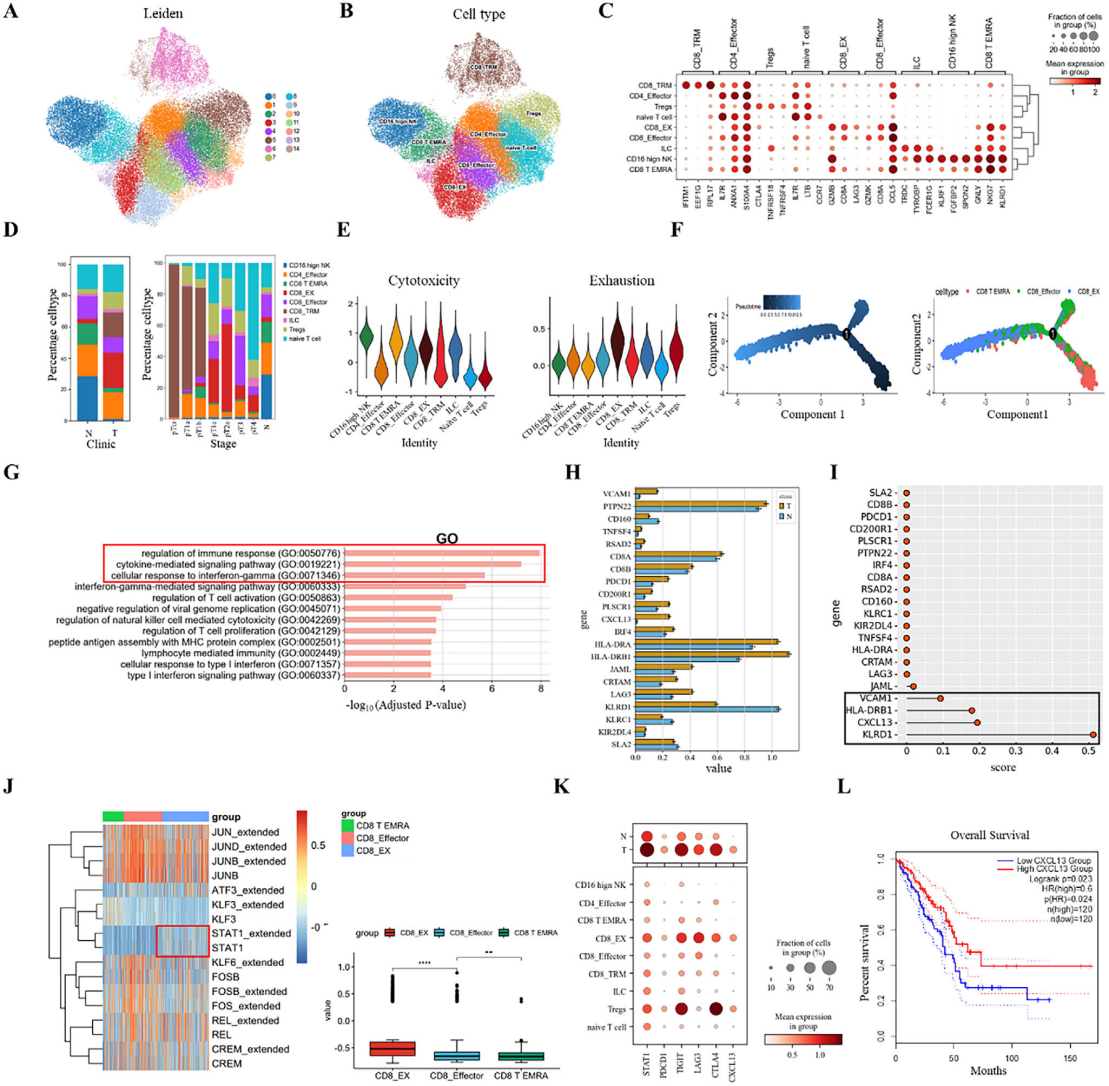
Single‐cell analysis uncovers the functional diversity and exhaustion signatures of T and NK cells in LUAD. A,B) UMAP visualization of T/NK cells showing unsupervised clustering (A) and cell type annotation based on marker gene expression (B), identifying nine subsets: CD8_TRM, CD8_EX, CD8^+^ effector, CD8^+^ TEMRA, CD4^+^ effector, Tregs, naive T cells, CD16 high NK cells, and ILC. C) Dot plot illustrating the expression of representative marker genes across T/NK cells subsets. D) Relative proportions of four T/NK cells subsets across samples stratified by tissue origin (left) and pT stage (right). E) Violin plots showing cytotoxicity and exhaustion scores among T/NK cells subsets. F) Pseudotime trajectory analysis constructed using Monocle 2 reveals the differentiation landscape of CD8 T cells subsets. Cells are colored according to pseudotime values (left) and annotated CD8 T cell subtypes (right), respectively. G) GO enrichment analysis of 346 genes upregulated in CD8⁺ exhausted T cells. H) The bar plot displays the expression levels of differentially upregulated genes in CD8⁺ EX cells across tumor (T, orange) and normal (N, blue) tissues. I) Feature importance ranking of top discriminatory genes identified via decision tree analysis. J) The most differentially enriched transcription factors across three CD8⁺ T cell subtypes by SCENIC algorithm (left), the transcription factors exclusively enriched in CD8⁺ EX cells are highlighted with red boxes. Box plot comparing STAT1 expression level across CD8⁺ T cell subtypes (right). K) Dot plot presenting the expression of representative immune checkpoint genes across various T/NK cells subsets. Dot size reflects the fraction of cells expressing each gene, and color intensity indicates average expression. L) Kaplan–Meier survival analysis comparing TCGA LUAD patients with high versus low CXCL13 expression. *P‐*value calculated using the two‐sided log‐rank test. **P < 0.01, ****P < 0.0001.

T lymphocytes serve as pivotal mediators of anti‐tumor immunity and represent key targets for immune checkpoint blockade therapies.^[^ ^16^ ^]^ Using the Leiden clustering algorithm, we identified fifteen cell subclusters of T cells and NK cells (**Figure** **4**A). These clusters were annotated into nine T/NK cell subtypes based on canonical marker genes, including CD8^+^ tissue‐resident memory T cells (CD8_TRM), exhausted CD8^+^ T cells (CD8_EX), CD8^+^ effector T cells, CD8^+^ TEMRA, CD4^+^ effector T cells, regulatory T cells (Tregs), naive T cells, CD16 high NK cells, and innate lymphoid cells (ILC) (Figure 4B). Dot plot analysis revealed marker expression patterns in nine T/NK cell subtypes (Figure 4C). Compared with early‐stage LUAD (pTis–pT1b), advanced disease stages (pT1c–pT4) exhibited increased frequencies of Tregs, CD8⁺ EX, and CD8‐effector subsets, suggesting stage‐associated remodeling of the T cell compartment (Figure 4D). Cytotoxic and exhaustion scores were quantified using the R package escape (v1.8.0) with established gene signatures (GZMB, PRF1, GNLY for cytotoxicity; PDCD1, LAG3, HAVCR2 for exhaustion). CD16 high NK cells and CD8⁺_TRM T cell clusters exhibited significantly higher cytotoxic scores compared to other subsets, whereas Tregs and CD8⁺_EX T cells exhibited high exhaustion scores (Figure 3E). Pseudotime analysis revealed a dynamic evolution of CD8⁺ T cell states during LUAD progression. CD8⁺_TEMRA T cells mapped to early disease stages, CD8⁺ effector T cells occupied an intermediate transitional state, and CD8⁺_EX T cells localized predominantly to terminal trajectory stages (Figure 4F). GO enrichment analysis of 346 differentially upregulated genes in CD8⁺_EX T cells (–log10 *p*‐value > 1.0, *p* < 0.05) identified significant enrichment in biological processes associated with immune response regulation, cytokine‐mediated pathways, and cellular response to interferon‐gamma (Figure 4G). Additionally, the expression profiles of these genes in LUAD and normal tissues were shown (Figure 4H). A decision tree–based feature importance analysis prioritized KLRD1, CXCL13, HLA‐DRB1, and VCAM1 as top‐ranked genes (*p* < 0.01) (Figure 4I). Integrated SCENIC and GSVA analyses identified enrichment of STAT1 regulatory motifs within CD8⁺_EX T cells transcriptional networks, suggesting potential STAT1‐mediated modulation of exhaustion‐related programs (Figure 4J). We further found that CD8‐EX T cells and Tregs exhibited notably high expression of multiple checkpoint markers, including PDCD1, TIGIT, LAG3, CXCL13, and CTLA4 (Figure 4K). Clinically, patients with elevated expression of CXCL13 was associated with improved survival in TCGA databases (HR = 0.6, *p* = 0.024; Figure 1L). Collectively, these results reveal a stage‐dependent reconfiguration of the T and NK cell landscape in LUAD, characterized by progressive enrichment of exhausted CD8⁺ T cells and immunosuppressive Tregs. STAT1 was identified as a potential transcriptional modulator of CD8⁺ T cell exhaustion, underscoring its possible relevance for immunomodulatory strategies.

### 2.5 Tumor‐Infiltrating B Cell Heterogeneity and Functional Dynamics in LUAD

**Figure 5:**
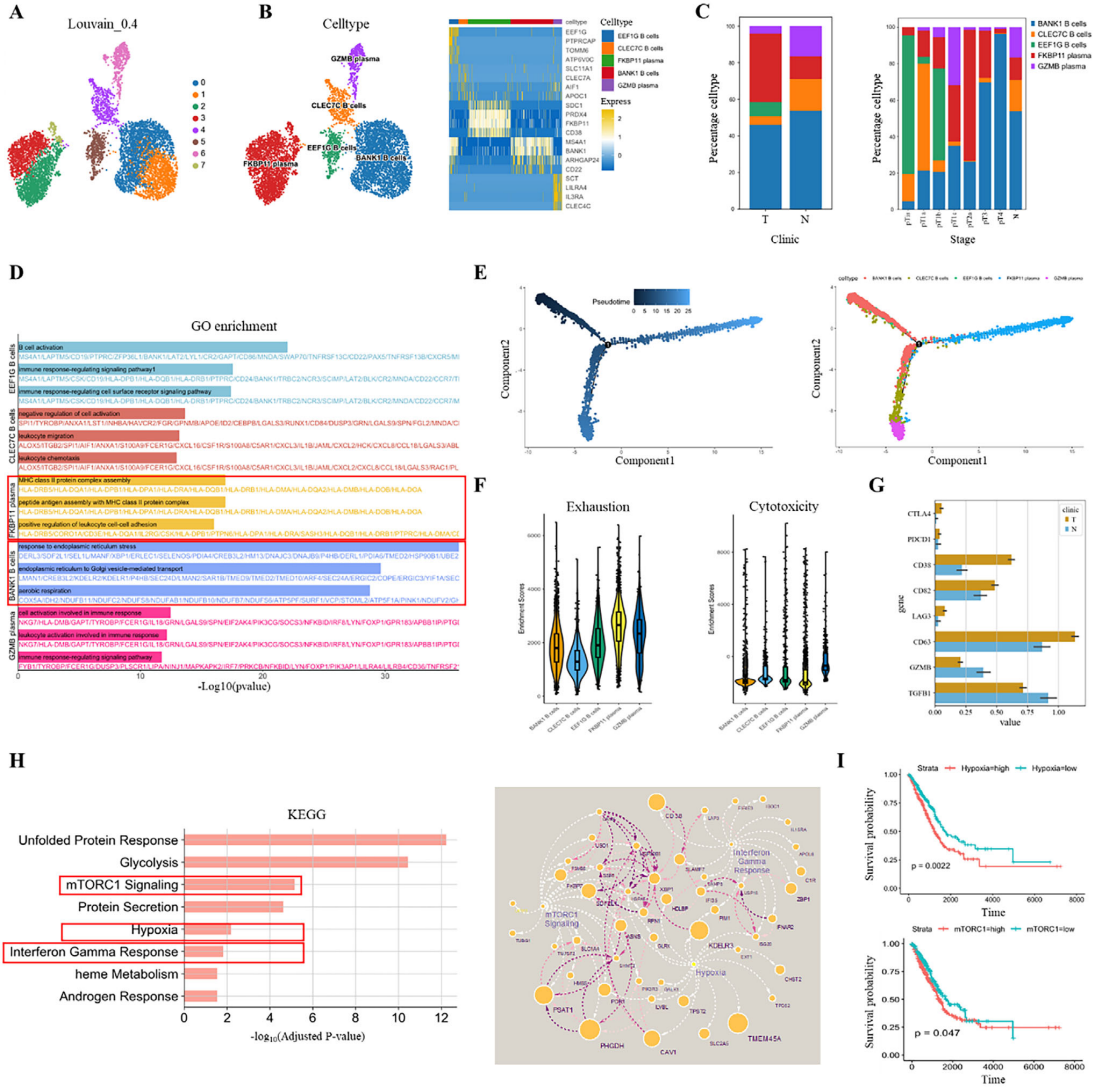
Single‐cell analysis characterizes the B cell subset heterogeneity and immune features of B cells in LUAD. A,B) UMAP visualization of B cells showing unsupervised clustering (A) and cell type annotation based on marker gene expression (B), identifying five subsets: EEF1G⁺ B cells, CLEC7C⁺ B cells, FKBP11⁺ plasma cells, BANK1⁺ B cells, and GZMB⁺ plasma cells (left). Heatmap showing marker genes for each B cell subcluster (right). C) Relative proportions of B cell subclusters across samples stratified by tissue origin (Left) and pathological T stage (right). D) GO enrichment analysis of DEGs across five major B cell subtypes. E) Pseudotime trajectory analysis constructed using Monocle 2 reveals the differentiation landscape of B cells subsets. Cells are colored according to pseudotime values (left) and annotated B cells subtypes (right), respectively. F) Violin plots showing cytotoxicity and exhaustion scores among B cells subsets. Each black dot corresponds to an individual patient. G) Bar plots showing the expression levels of immune checkpoint genes in FKBP11⁺ plasma cells derived from tumor and normal tissues. H) KEGG enrichment analysis of DEGs in FKBP11 plasma cells (left), PPI network depicting key proteins and interactions among the mTOR signaling, hypoxia, and interferon‐gamma response pathways (right). Network nodes represent proteins enriched in KEGG pathways. I) Kaplan–Meier survival analysis comparing TCGA LUAD patients with high versus low expression of the hypoxia gene set (top) and the mTORC1 gene set (bottom). *P*‐values were calculated using the two‐sided log‐rank test.

Recently, tumor‐infiltrating B cells have been recognized as integral immunoregulatory components within the TME, rather than merely passive auxiliary immune cells.^[^ ^17^ ^]^ Their functional heterogeneity and differentiation trajectories play pivotal roles in modulating anti‐tumor immune responses, predicting responses to immunotherapy, and determining clinical prognosis. In this study, we performed unsupervised clustering of B cells (**Figure** **5**A) and identified eight distinct subsets, which were annotated as EEF1G⁺ B cells, CLEC7C⁺ B cells, FKBP11⁺ plasma cells, BANK1⁺ B cells, and GZMB⁺ plasma cells based on canonical markers (Figure 5B). Notably, FKBP11⁺ plasma cells were significantly enriched in LUAD tissues compared to normal lung samples, suggesting tumor‐associated expansion. Furthermore, stage‐specific analysis revealed a marked accumulation of FKBP11⁺ plasma cells and BANK1⁺ B cells in advanced LUAD (pT1c–pT4), with BANK1⁺ B cells exhibiting peak abundance at the pT3–pT4 stages, indicating dynamic shifts in B cell composition during disease progression (Figure 5C). GO enrichment analysis revealed distinct functional specialization among these B cell subsets. FKBP11⁺ plasma cells were significantly enriched in antigen processing and MHC class II‐mediated presentation pathways, suggesting potential involvement in T cell activation. In contrast, BANK1⁺ B cells exhibited strong enrichment in endoplasmic reticulum (ER) stress responses and secretory vesicle transport pathways, consistent with enhanced antibody secretion capacity. Meanwhile, GZMB⁺ plasma cells were linked to immune effector activation and NF‐κB signaling, suggesting involvement in cytotoxic immune responses (Figure 5D). Pseudotemporal trajectory analysis revealed a unidirectional differentiation continuum of B cells in LUAD. FKBP11⁺ and GZMB⁺ plasma cells were found to occupy separate terminal branches, suggesting that they represent two functionally distinct terminal states (Figure 5E). Using the R package escape (v1.8.0) and established gene signatures (GZMB, PRF1, GNLY for cytotoxicity; PDCD1, LAG3, HAVCR2 for exhaustion), we quantified the cytotoxic and exhaustion scores. FKBP11⁺ plasma cells exhibited significantly elevated exhaustion scores (Figure 5F), accompanied by a coordinated upregulation of key immune checkpoint regulators (CTLA4, PDCD1, CD38, and LAG3). In contrast, the expression of the cytotoxic effector molecule GZMB was markedly downregulated (Figure 5G). Moreover, KEGG and PPI analysis indicated enrichment of pathways related to mTORC1 signaling, hypoxia response, and interferon‐gamma response, with key metabolic regulators such as PHGDH, PSAT1, PDK1, RPS6, and SHMT2 highly expressed (Figure 5H). Clinically, analysis of the TCGA LUAD cohort revealed that high expression of hypoxia and mTORC1 gene sets was significantly associated with poor overall survival (*p* = 0.0022 and *p* = 0.047, respectively) (Figure 5I). These findings reveal a functionally heterogeneous landscape of tumor‐infiltrating B cells in LUAD, highlighting FKBP11⁺ plasma cells as a dominant immunosuppressive population and a potential target for immunometabolic therapy.

### 2.6 Single‐Cell Transcriptomic Profiling Reveals Heterogeneous CAF Subsets in Lung Adenocarcinoma

**Figure 6:**
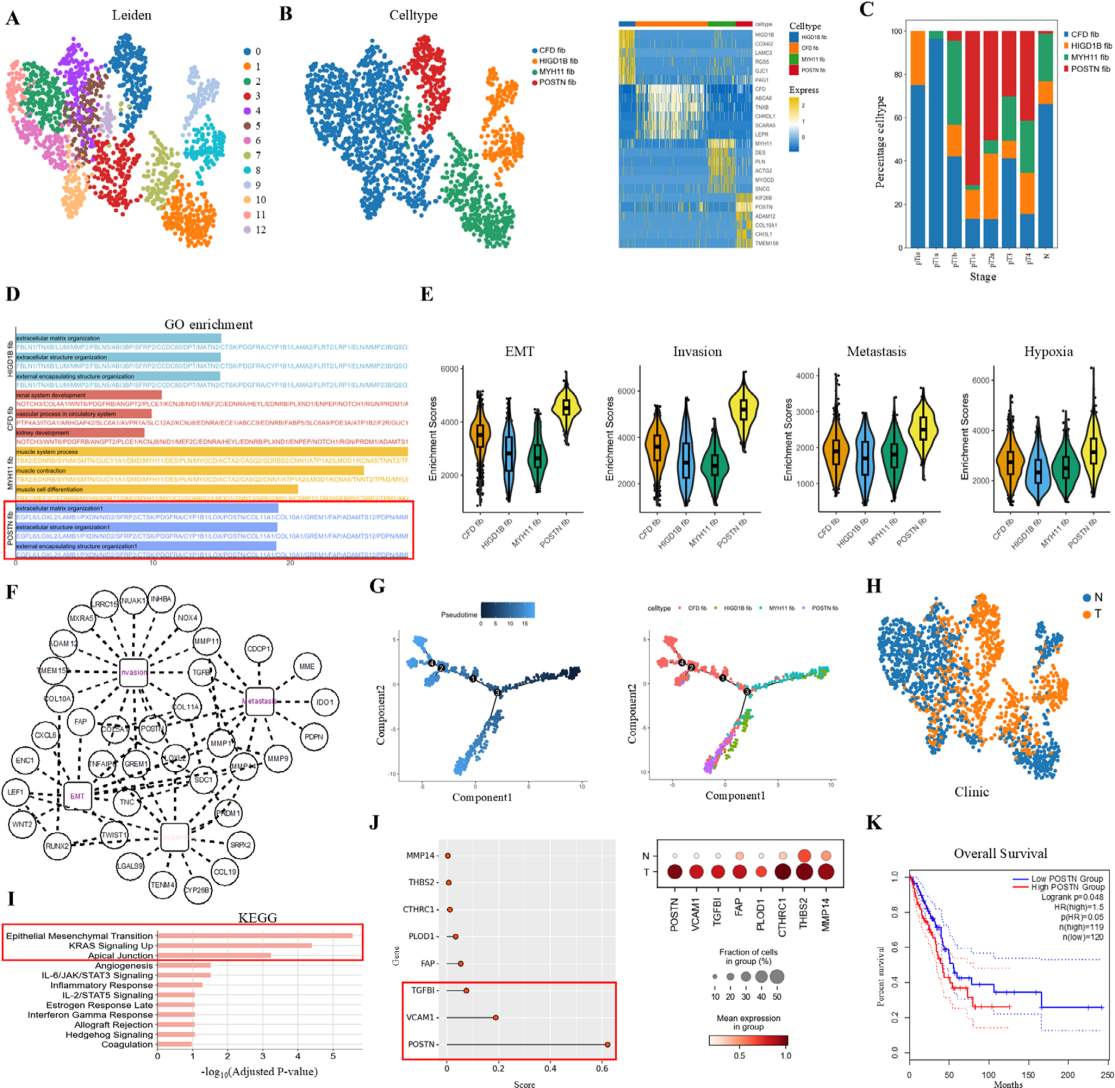
Single‐cell transcriptional analysis reveals heterogeneity of fibroblasts in LUAD. A,B) UMAP visualization of fibroblasts showing unsupervised clustering (A) and cell type annotation based on marker gene expression (B), identifying four subsets: HIGD1B^+^ fibroblasts, CFD^+^ fibroblasts, MYH11^+^ fibroblasts, and POSTN^+^ fibroblasts (left). Heatmap showing the marker genes in four fibroblast subtypes (right). C) Relative proportions of four fibroblast subsets across LUAD progression stages. D) GO enrichment analysis of DEGs across four major fibroblasts subtypes. E) Violin plots showing EMT, invasion, metastasis, and hypoxia enrichment scores among fibroblasts subsets. Each black dot corresponds to an individual patient. F) PPI network illustrating key proteins involved in EMT, invasion, metastasis and hypoxia processes and their interaction networks. G) Pseudotime trajectory analysis constructed by Monocle 2 reveals the differentiation landscape of fibroblasts subsets. Cells are colored according to pseudotime values (left) and annotated fibroblast subtypes (right), respectively. H) UMAP plots showing the distribution of fibroblasts in normal and tumor samples. I) KEGG enrichment analysis of upregulated genes in tumor‐derived fibroblasts. J) Feature importance ranking of genes involved in POSTN⁺‐associated oncogenic pathways in LUAD based on a decision tree model (left), and Dot plot presenting the expression of representative genes in normal and tumor tissues (right). Dot size reflects the fraction of cells expressing each gene, and color intensity indicates average expression. K) Kaplan–Meier survival analysis of TCGA LUAD patients stratified by high versus low POSTN expression. *P*‐value calculated using a two‐sided log‐rank test.

Cancer‐associated fibroblasts (CAFs) are critical regulators of tumor progression, contributing to ECM remodeling, immunosuppression, and resistance to therapy.^[^ ^18^ ^]^ We identified thirteen distinct CAF subclusters (C0–C12) (**Figure** **6**A), which were further classified into four distinct subtypes based on marker gene expression: HIGD1B^+^ fibroblasts, CFD^+^ fibroblasts, MYH11^+^ fibroblasts, and POSTN^+^ fibroblasts (Figure 6B). Comparative analysis revealed significant enrichment of POSTN^+^ fibroblasts in advanced‐stage lung adenocarcinoma (pT1c‐pT4) relative to early‐stage disease (pTis–pT1b) (Figure 6C). GO enrichment analysis indicated that POSTN⁺ fibroblasts were enriched in extracellular matrix reorganization and structural modulation of encapsulating components (Figure 6D). Pathway activity scoring revealed enrichment of EMT, invasion, metastasis, and hypoxia‐related pathways in POSTN⁺ fibroblasts, suggesting potential involvement in tumor‐promoting processes (Figure 6E). PPI network analysis further identified four core functional modules within the POSTN⁺ fibroblast subset, with metastasis/invasion‐related genes (COL11A1, LRRC15, MMP1) showing particularly elevated expression (Figure 6F). Pseudotemporal trajectory analysis uncovered two distinct differentiation pathways originating from HIGD1B^+^ and MYH11^+^ subclusters, ultimately diverging into POSTN^+^ and CFD^+^ terminal states (Figure 6G). After distinguishing the tumor from the normal subgroup, comparative KEGG pathway analysis revealed LUAD‐specific enrichment in EMT, KRAS signaling, and apical junction pathways (Figure 6H,I). Decision tree modeling identified POSTN, VCAM1, and TGFBI as top‐ranked discriminative biomarkers across four POSTN^+^‐associated oncogenic pathways in LUAD tissues (p < 0.01) (Figure 6J). Clinically, elevated POSTN expression was significantly associated with reduced overall survival in LUAD patients based on TCGA dataset analysis (*p* = 0.05) (Figure 6K). These results define a functionally specialized and transcriptionally heterogeneous CAF landscape in LUAD, with POSTN⁺ fibroblasts representing a transcriptionally distinct subset associated with tumor‐promoting and potential therapeutic targets for stroma‐directed interventions.

## 3 Discussion

In the present study, we revealed coordinated remodeling across malignant, immune, and stromal compartments during LUAD progression, uncovering how the tumor microenvironment transitions from an immune‐activated state to a hypoxia‐enriched, immunosuppressive ecosystem. Early‐stage LUAD was characterized by highly proliferative epithelial cells and preserved immune surveillance, whereas advanced stages exhibited enhanced EMT and metabolic adaptation. Concomitantly, the immune and stromal compartments underwent extensive reprogramming, marked by the accumulation of cell subsets that collectively attenuate anti‐tumor immunity and promote a tumor‐supportive microenvironment.

Tumor epithelial cells exhibited progressive heterogeneity and functional reprogramming with disease progression.^[^ ^19^ ^]^ CNV‐based inference identified 12566 malignant cells, which were classified into seven transcriptionally distinct subsets. Among these, the C5 subcluster was predominantly enriched in advanced‐stage LUAD (pT3–pT4) and showed pronounced activation of EMT, invasion, metastasis, and hypoxia‐associated transcriptional programs. These findings are consistent with previous studies suggesting that EMT and hypoxic signaling synergistically promote malignant progression and therapeutic resistance in LUAD.^[^ ^20^, ^21^ ^]^ Within this subset, key effectors such as MMP11 and MMP13 were upregulated, consistent with roles in extracellular matrix remodeling and invasive behavior, while canonical HIF‐1α downstream targets, including STC2, ALDOC, and SPAG4, suggested transcriptional adaptation to hypoxic stress.^[^ ^22^, ^23^ ^]^ Importantly, the upregulation of hypoxia‐associated genes such as STC2, AK4, PFKP, and STC1 was significantly correlated with poor clinical outcomes, reflecting enhanced glycolysis, mitochondrial maintenance, and cellular plasticity under hypoxic conditions.^[^ ^22^ ^]^ In our analysis, elevated expression of these genes also emerged as independent unfavorable prognostic biomarkers in LUAD, underscoring their clinical relevance. Collectively, these results suggest that the C5 tumor cell subset may represent a hypoxia‐adapted epithelial phenotype with high metastatic potential and clinical relevance.

TAMs are the most prominent immune cell population in the TME and play a critical regulatory role in the progression of LUAD, exhibiting pronounced functional heterogeneity and adopting diverse phenotypes ranging from pro‐inflammatory to immunosuppressive.^[^ ^24^ ^]^ In this study, LGMN⁺ and FCN1⁺ MAC subsets were identified as being closely associated with LUAD progression. LGMN⁺ MACs were significantly enriched in advanced LUAD stages (pT1c–pT4), exhibited transcriptional features consistent with an M2‐like immunosuppressive phenotype, and were localized to the terminal stages of the pseudotime developmental trajectory. Previous studies have demonstrated that hypoxia is a major driver of the immunosuppressive TAM phenotype within the TME.^[^ ^25^, ^26^ ^]^ Notably, HIF‐1α has been reported to regulate LGMN transcription, thereby facilitating M2‐like polarization and enhancing tumor‐supportive functions in macrophages.^[^ ^27^ ^]^ Consistently, our data revealed a hypoxia‐enriched malignant epithelial subset (C5) that was predominantly upregulated in late‐stage LUAD, implicating the hypoxia–HIF‐1α–LGMN axis as a potential regulatory pathway contributing to immunosuppressive TME remodeling and disease progression. In addition, FCN1 has been identified as a key marker of pro‐inflammatory monocyte‐derived macrophages.^[^ ^28^ ^]^ However, emerging evidence suggested that FCN1⁺ monocytes may act as precursors of C1Q⁺ immunosuppressive TAMs, a subset characterized by the expression of multiple immunosuppressive genes and contributing to immunosuppression.^[^ ^29^ ^]^ These findings underscore the phenotypic plasticity and functional diversity of LGMN⁺ and FCN1⁺ TAM subsets, and highlight their potential as therapeutic targets for reprogramming the immunosuppressive microenvironment and enhancing antitumor immunity in LUAD.

T cells and NK cells are critical effector populations involved in tumor immune surveillance and anti‐tumor immunity. During the progression of LUAD, this process is frequently accompanied by a gradual decline in the proportion of NK cells, along with a marked accumulation of exhausted T cells and Tregs.^[^ ^7^ ^]^ Consistent with previous studies, we observed that tumor progression is associated with immunosuppression and T cell exhaustion, suggesting that the TME in advanced‐stage LUAD is characterized by a shift toward an “immunosuppressive‐exhausted” state. Importantly, although CD8⁺EX cells exhibited typical transcriptional features of exhaustion, our analysis revealed retention of transcriptional activity related to TCR signaling and cytokine‐mediated regulatory pathways. This suggests that CD8⁺EX cells are not entirely functionally silent and may retain partial effector functionality, thus representing a population with potential responsiveness to immunotherapy. In particular, the chemokine CXCL13, a hallmark of tumor‐reactive T cells, was highly expressed in this subset and has been previously correlated with favorable responses to ICB, supporting its potential prognostic and therapeutic relevance.^[^ ^30^ ^]^ Feature‐ranking analysis further identified VCAM1, HLA‐DRB1, CXCL13, and KLRD1 as core markers of CD8⁺EX cells, supporting their immunomodulatory heterogeneity and clinical relevance. Notably, VCAM1 and HLA‐DRB1, involved in cell adhesion and antigen presentation, respectively, suggest that CD8⁺EX cells retain immune interactive capacity and may represent potential biomarkers or targets for combinatorial immunotherapy. Furthermore, using the SCENIC algorithm, we inferred transcriptional regulatory networks and identified STAT1 as a potential upstream regulator driving the exhausted phenotype in CD8⁺ T cells. SCENIC has been widely validated for its ability to robustly reconstruct gene regulatory networks from single‐cell transcriptomic data and accurately infer transcription factor activity at the cellular level.^[^ ^18^, ^31^, ^32^ ^]^ The consistency between our SCENIC‐based predictions and known STAT1‐mediated immunoregulatory pathways further supports the reliability of our analysis.^[^ ^33^ ^]^ Given that hypoxia can activate STAT1 through multiple pathways, this mechanism may further exacerbate T cell exhaustion and immune suppression. These findings suggest that LUAD progression may involve STAT1‐mediated transcriptional reprogramming of T cells toward an exhausted and immunoregulatory state, and that CXCL13 expression might indicate potential responsiveness to immune checkpoint blockade therapy.

Within the tumor microenvironment, B cells are increasingly recognized as susceptible to functional exhaustion, a phenomenon that becomes more pronounced during tumor progression. Although the characterization of B cell exhaustion remains less extensively studied than that of T cells, accumulating evidence has demonstrated that tumor‐infiltrating B cells can acquire transcriptional features associated with dysfunctional states. Specifically, activated memory B cells have been shown to upregulate immune checkpoints (PDCD1, CD274, CTLA4, ENTPD1, LAG3, and HAVCR2) and exhaustion‐associated TFs (TOX, TOX2, ZBED2, BATF, RBPJ, and VDR), thereby impairing their capacity for antibody production.^[^ ^17^ ^]^ Here, we identified five phenotypically and functionally distinct B cell subsets, EEF1G⁺ B cells, CLEC7C⁺ B cells, FKBP11⁺ plasma cells, BANK1⁺ B cells, and GZMB⁺ plasma cells. Among these, FKBP11⁺ plasma cells exhibited hallmark features of immune exhaustion, including elevated expression of multiple immune checkpoint regulators (PDCD1, CTLA4, LAG3) and concomitant downregulation of cytotoxic effector molecules such as GZMB. Importantly, this subset was transcriptionally enriched for hypoxia‐associated metabolic reprogramming genes (PHGDH, PDK1, SHMT2) downstream of HIF‐1α, implicating a role for hypoxic signaling in shaping their exhausted phenotype.^[^ ^34^, ^35^ ^]^ Thus, our results underscore that hypoxia‐driven metabolic reprogramming may contribute to the functional exhaustion of tumor‐infiltrating B cells, particularly FKBP11⁺ plasma cells, thereby linking metabolic stress to immune suppression and tumor progression in LUAD.

In parallel, we characterized the pronounced heterogeneity of CAFs in LUAD and identified a distinct POSTN⁺ CAF subset with pronounced protumorigenic potential and microenvironmental adaptability. Consistent with previous studies implicating POSTN in ECM remodeling, tumor invasion, and immune evasion across various malignancies,^[^ ^36^, ^37^, ^38^ ^]^ our data showed a significant enrichment of POSTN⁺ CAFs in advanced‐stage LUAD. Pseudotime trajectory analysis further suggested that POSTN⁺ CAFs may arise from HIGD1B⁺ and MYH11⁺ precursor lineages, representing a terminally differentiated CAF phenotype with enhanced functional specialization. Functional enrichment analysis further showed that POSTN⁺ CAFs were enriched for hypoxia‐adaptive programs, with upregulation of hallmark pathways such as EMT, KRAS signaling, and angiogenesis, suggesting that this subset may possess enhanced survival and protumor potential in hypoxic tumor niches. This observation is supported by previous reports showing that hypoxia can directly induce POSTN expression and secretion in CAFs.^[^ ^39^ ^]^ Moreover, elevated expression of TGFBI and VCAM1 within POSTN⁺ CAFs suggests potential roles in mediating tumor cell adhesion, migration, and immunomodulation. These molecules may contribute to the establishment of an immune‐excluded phenotype by enhancing ECM density, forming physical barriers to immune infiltration, and modulating adhesion and chemotactic signaling cues. Collectively, these findings suggest that POSTN⁺ CAFs, particularly under hypoxic conditions, may function as potential coordinators of ECM remodeling, metabolic adaptation, and immune suppression.

**Figure 7:**
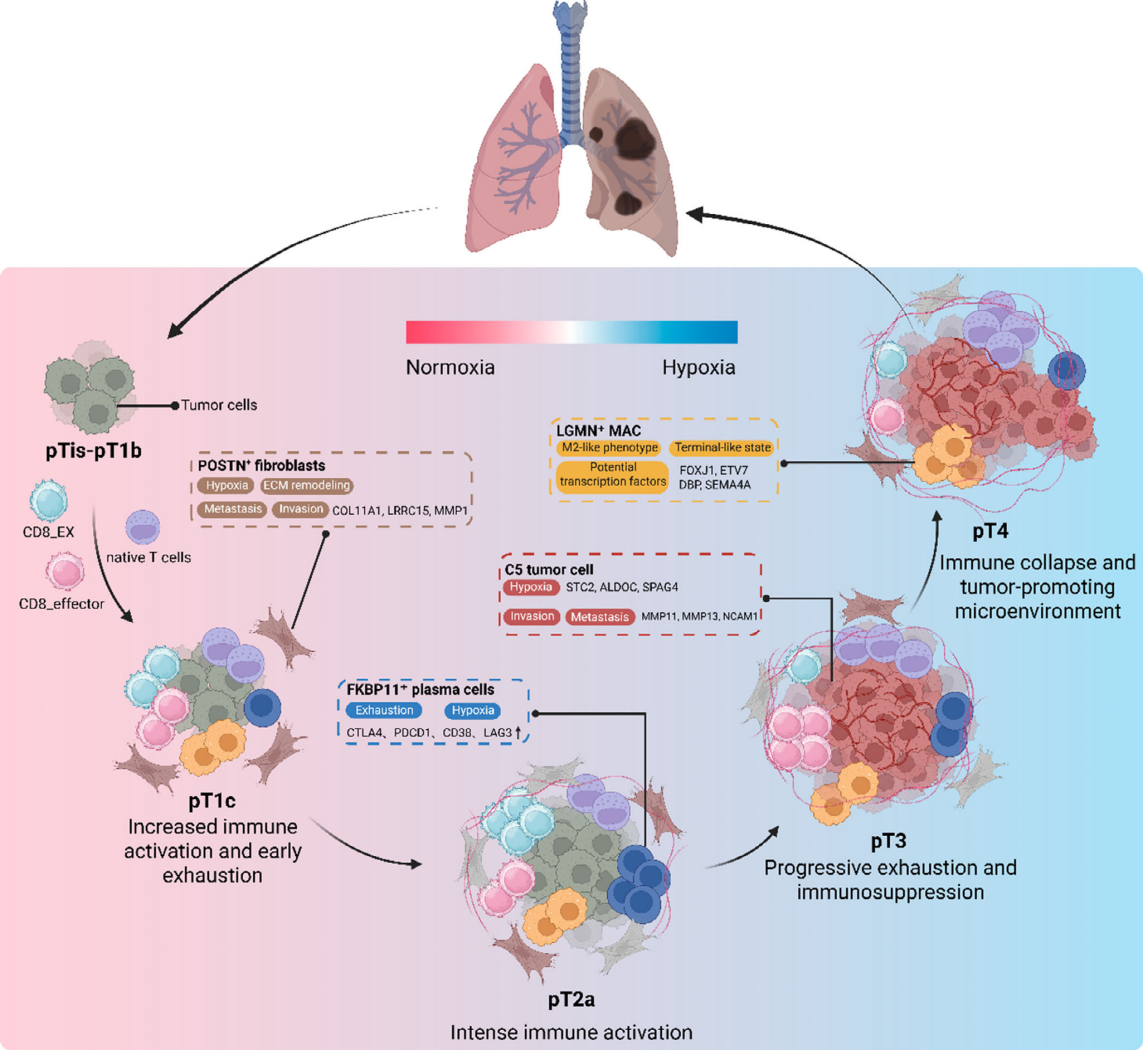
Schematic overview illustrating the stage‐specific cellular remodeling and hypoxia‐driven evolution during LUAD progression. In early lesions (pTis‐pT1b), the microenvironment remains largely normoxic with minimal immune engagement. At pT1c, immune activation increases, accompanied by early exhaustion and the appearance of POSTN^+^ fibroblasts with ECM‐remodeling signatures. By pT2a, heightened immune activity coincides with emerging dysfunction and the expansion of FKBP11^+^ plasma cells expressing exhaustion‐ and hypoxia‐associated markers. As tumors progress to pT3, rising hypoxia promotes the emergence of a C5 malignant subset enriched for invasion and metastasis programs, together with exacerbated immune dysfunction. In advanced disease (pT4), severe hypoxia leads to widespread immune collapse, characterized by the accumulation of immunosuppressive LGMN^+^ macrophages, collectively establishing a tumor‐promoting microenvironment. Created with BioRender.com.

In conclusion, we identified a hypoxia‐enriched malignant epithelial subset (C5) that was markedly expanded in advanced‐stage LUAD and transcriptionally enriched in EMT‐ and metastatic‐associated programs. Within the TAM compartment, LGMN⁺ macrophages exhibited a distinctly immunosuppressive phenotype, whereas FCN1⁺ macrophages demonstrated greater functional plasticity, indicating divergent TME remodeling. CD8⁺EX T cells displayed hallmark features of dysfunction but retained partial immune responsiveness, potentially under STAT1‐mediated regulation. Among B cells, FKBP11⁺ plasma cells exhibited a canonical exhausted phenotype and were transcriptionally enriched for HIF‐1α‐associated metabolic reprogramming signatures. In the stromal compartment, CAFs showed pronounced heterogeneity, with the POSTN⁺ subset representing a terminally differentiated CAF population enriched for hypoxia‐adaptive programs, suggesting potential roles in ECM remodeling and immunosuppression (Figure **7**). Collectively, these findings suggest that the interplay between metabolic adaptation and immune regulation constitutes a central axis of LUAD progression and immune evasion, providing a framework to guide strategies aimed at restoring immune competence within the LUAD microenvironment. Although the present cohort effectively captures key phenotypic and transcriptional features associated with disease advancement, future studies with larger and diverse clinical subtypes cohorts, complemented by functional validation using in vitro and in vivo models, will be essential to substantiate these mechanisms and enhance the translational relevance of these findings.

## 4 Experimental Section

### Ethics Statement and Patient Samples

This study was approved by the Ethics Committee of The Third People's Hospital of Chengdu (Approval No. 2023‐S‐203), and procedures were conducted in accordance with the Declaration of Helsinki and relevant ethical guidelines. All subjects have provided their written informed consent. A total of six early‐stage LUAD specimens were obtained from patients with CT‐confirmed pulmonary tumors smaller than 2 cm in diameter. Fresh tissue samples of lung lesions were directly collected in the operating room.

### Single‐Cell Suspension Preparation

Single‐cell suspensions were prepared from lung tissues as previously described.^[^ ^40^ ^]^ Briefly, fresh tissues were immediately transferred into ice‐cold RPMI 1640 medium (Gibco) supplemented with 10% heat‐inactivated fetal bovine serum (Gibco). Tissues were then minced into small fragments and enzymatically digested in a dissociation solution containing neutral protease, collagenase types I, II, and IV, and DNase I at 37 °C for 1 hour with gentle agitation on a shaker. The resulting cell suspensions were then filtered through a 70 µm cell strainer (Falcon), and centrifuged at 400 × g for 5 minutes. The cell pellets were resuspended in red blood cell lysis buffer (BD Biosciences) to eliminate erythrocytes, washed, and subsequently resuspended in Dulbecco's Modified Eagle Medium (DMEM, Gibco) containing 5% FBS. Cell counts and viability were subsequently assessed to ensure high‐quality single‐cell preparations for downstream analyses.

### Library Preparation and scRNA‐Seq

Single‐cell RNA sequencing was performed using the 10 × Genomics Chromium platform following the manufacturer's protocol. Briefly, prepared cell suspensions were loaded onto a Chromium Next GEM Chip along with 10 × barcoded gel beads and partitioning oil to generate Gel Beads‐in‐Emulsion (GEMs) using a microfluidic encapsulation system. Within each GEM, individual cells were lysed, and the released mRNA transcripts were hybridized to barcoded oligonucleotide primers attached to the gel beads. After reverse transcription, GEMs were disrupted, and the barcoded cDNA was purified, amplified, and subjected to standard library preparation workflows, including fragmentation, end repair, A‐tailing, adaptor ligation, and PCR enrichment. The final libraries were sequenced on an Illumina NovaSeq 6000 system using 150 bp paired‐end reads, targeting a sequencing depth of at least 100000 reads per cell.

### Single‐Cell RNA Sequencing Data Collection

This study incorporated a publicly available scRNAseq dataset of LUAD from https://doi.org/10.24433/CO.0121060.v1 (P018, P019, P023, P024, P027, P028, P029, P030, P031, P032, P033, P034)  and performed integrated analyses in combination with six newly generated early‐stage LUAD specimens (P01, P02, P03, P04, P05, P06).^[^ ^41^ ^]^ This dataset was selected as the late‐stage cohort because it provides a comprehensive and well‐annotated single‐cell transcriptomic resource of advanced LUAD, generated using the 10 x Genomics platform with standardized sequencing quality and detailed clinical annotation. Tumor staging for all samples was determined according to the Tumor‐Node‐Metastasis (TNM) classification system established by the American Joint Committee on Cancer (AJCC, 8th edition).

### Single‐Cell RNA Sequencing Data Processing

The Cell Ranger toolkit (version 7.0.1) provided by 10 × Genomics was utilized to align and annotate reads using human genome assembly GRCh38. The resulting gene‐barcode matrices were imported into R (version 3.6.0) and analyzed using the Seurat package (version 4.1.0). High‐quality cells (500–5000 genes, 1600–25000 UMIs, and mitochondrial content less than 15%) were selected for further analyses.^[^ ^42^ ^]^ Doublets were identified and removed using the DoubletFinder package. After quality control and filtering, a final single‐cell dataset comprising 123725 cells from 18 patients was obtained for subsequent analyses.

### Unsupervised Dimensional Reduction and Identification of Major Cell Clusters

To identify major cell populations, normalization of the integrated dataset was performed using the NormalizeData function in the Seurat package. Principal component analysis (PCA) was then applied for linear dimensionality reduction based on highly variable genes. The Harmony algorithm, widely used for batch effect correction in single‐cell transcriptomic studies,^[^ ^43^ ^]^ was applied to integrate the datasets with the publicly available datasets. This approach minimized technical variation while preserving biological heterogeneity for downstream analyses. Following dimensional reduction, all cells were embedded in a 2D space using UMAP, enabling the clustering of cells with similar transcriptional profiles. DEGs for each cluster were identified using the FindAllMarkers function in Seurat, and cell types were annotated based on the expression of canonical marker genes. Malignant epithelial cells were further distinguished from non‐malignant counterparts by inferring large‐scale CNVs using the InferCNV algorithm. Immune cell subtypes, including macrophages, CD4 T cells, and CD8 T cells, were designated as the reference population to baseline‐correct somatic CNVs.

### Clustering and Annotation of Cell Sub‐Populations

To further delineate the intracluster heterogeneity within major cell types, a second round of unsupervised clustering was performed. Briefly, each major cell type was extracted from the annotated Seurat object and subjected to a standard preprocessing workflow, including data normalization, variable feature selection, data scaling, and PCA. A shared nearest neighbor graph was constructed and clustering was performed using a multiple resolution parameter ranging from 0.4 to 1.5,^[^ ^44^ ^]^ followed by UMAP embedding for visualization. Subcluster‐specific marker genes were identified using the *FindAllMarkers* function. Subsets were annotated based on the top DEGs, and named using the format “cluster_ marker_ celltype” to ensure clarity and consistency across datasets.

### Single‐Cell Differential Gene Expression Analyses and Functional Enrichment Analyses

Single‐cell differential gene expression analyses were performed using MAST R package.^[^ ^45^ ^]^ Likelihood ratio tests were performed between the full and reduced model formulas to identify DEGs. Multiple testing corrections were applied using the Benjamini–Hochberg method, and genes with an FDR < 0.05 were designated as DEGs. The FindMarkers and FindAllMarkers functions in Seurat were used to calculate cluster‐specific marker genes (min.pct = 0.3, logfc.threshold = 0.25). GO and KEGG enrichment analyses were conducted using the clusterProfiler package in R.

### Pseudotime Trajectory Analyses

Monocle R package v 2.20.0 was used for inferring the pseudotime trajectories.^[^ ^46^ ^]^ Pseudotime trajectories were constructed using high‐variability gene sets identified between cell clusters in Seurat. After dimensionality reduction with the DDRTree method, single cells were mapped onto trajectory trees, and pseudotime states were assigned using the orderCells function. Branch‐specific gene expression patterns were subsequently analyzed using the BEAM function.

### Machine Learning and Decision Tree

The expression matrices of key marker genes in cell subsets were standardized using the StandardScaler module from sklearn. Decision tree analysis was conducted with the SciKit‐Learn Python package, with gene expression features serving as input variables. The predictive performance was evaluated based on the area under the receiver operating characteristic curve (ROC‐AUC).

### Transcription Factor Analysis

Transcription factor activity analysis was performed using the SCENIC workflow to identify active transcription factor modules.^[^ ^31^ ^]^ Initially, the RcisTarget transcription factor motif database for the hg38 human reference genome was downloaded, and genes not present in the database were filtered out. Gene co‐expression modules were inferred using the GENIE3 R package. Transcription factor regulatory networks were reconstructed with the SCENIC R package. To evaluate regulon activity and visualize the results, the AUCell package in R was utilized.

### Survival Analyses

RNA‐seq and corresponding clinical data from LUAD patients were obtained from The Cancer Genome Atlas (TCGA) to evaluate the prognostic effects of gene sets derived from specific cell states. Overall survival was estimated using the Kaplan–Meier method with pairwise comparison. Patients were grouped based on specific characteristics, and survival curves were generated for each group, accounting for censored data. The log‐rank test was used to compare survival between groups and assess statistical significance. Median survival times were calculated to summarize the time until the event of interest occurred, identify factors associated with poor prognosis.

### Statistical Analysis

All statistical analyses were conducted using R software. Specific statistical methods applied to each dataset are detailed in the corresponding sections of the “Experimental Section”. The *p*‐values < 0.05 was considered statistically significant for all tests.

## Conflict of Interest

The authors declare no conflict of interest.

## Author Contributions

B.Q., X.L., and X.H. shared co‐first authorship and contributed equally to this work. B.M.Q., L.X.L., and X.H. conceptualized the idea and contributed to the design. J.Y.W., M.L.J., Y.Y., L.Z., B.B.Y., A.Y.X., and R.Q. performed data analysis. J.Y.W. and M.L.J. performed experiments. B.M.Q., L.X.L., X.H., and G.P.L. contributed to manuscript writing. All authors read and approved the final manuscript.

## Supporting information



Supporting Information

## Data Availability

The data that support the findings of this study are available from the corresponding author upon reasonable request.
