# Supplementary material for: Characterizing Stage‐Specific Cellular Dynamics and Microenvironmental Remodeling in Lung Adenocarcinoma by Single‐Cell RNA Sequencing
Source: Adv Sci (Weinh). 2025 Nov 28;13(9):e10847. doi: 10.1002/advs.202510847 (PMC12904057; doi:10.1002/advs.202510847)
Supplement: Supplementary file 1 — Supporting Information [file ADVS-13-e10847-s001.docx]

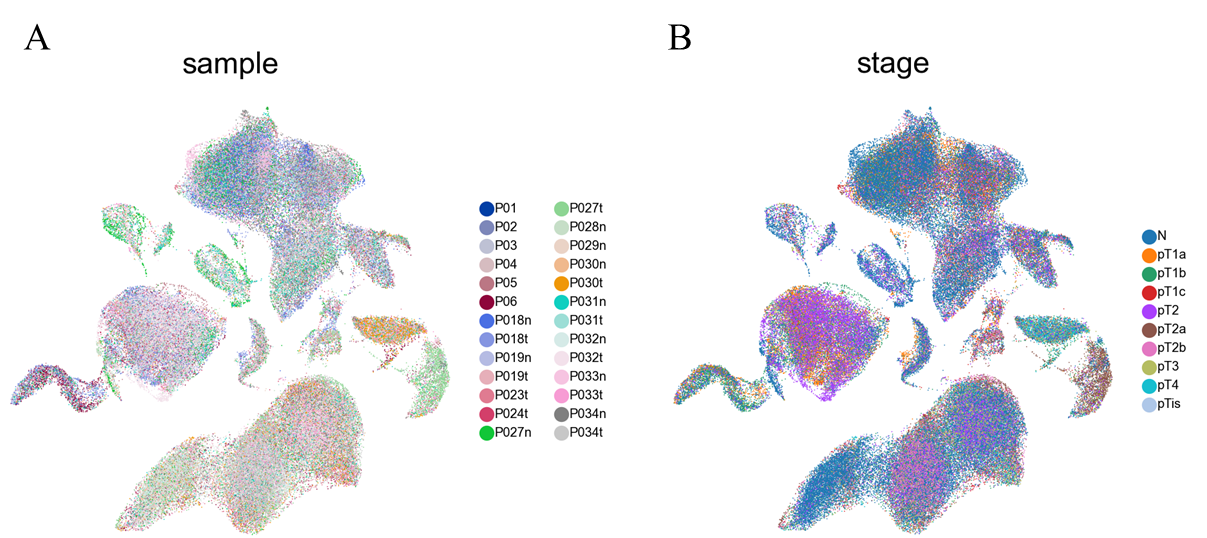


**Fig. S1. UMAP visualization of single-cell transcriptomes from LUAD and adjacent normal tissues.** (A) UMAP plot colored by individual samples. (B) UMAP plot colored by pathological stage, demonstrating the distribution of cells across disease progression from adenocarcinoma in situ (pTis) to stage pT4, with normal samples (N) indicated in blue.

| **Tissue types** | **Patient ID** | **Tissue origins** | **Topography** | **Tumor** | **Sex** | **Age** | **Origin** |
| --- | --- | --- | --- | --- | --- | --- | --- |
| Tumor | P01 | Lung | Upper lobe | pTis | Female | 57 | Local hospital |
|  | P02 | Lung | Upper lobe | pTis | Male | 52 |  |
|  | P03 | Lung | Upper lobe | pT1a | Female | 46 |  |
|  | P04 | Lung | Upper lobe | pT1b | Male | 57 |  |
|  | P05 | Lung | Upper lobe | pT1a | Female | 57 |  |
|  | P06 | Lung | Lower lobe | pT1a | Male | 67 |  |
|  | P018 | Lung | Upper lobe | pT1a | Female | 58 | Published dataset |
|  | P019 | Lung | Upper lobe | pT2a | Male | 81 |  |
|  | P023 | Lung | Upper lobe | pT1c | Female | 62 |  |
|  | P024 | Lung | Lower lobe | pT1b | Female | 63 |  |
|  | P027 | Lung | Upper lobe | pT2a | Male | 49 |  |
|  | P030 | Lung | Lower lobe | pT4 | Male | 63 |  |
|  | P031 | Lung | Lower lobe | pT2 | Male | 77 |  |
|  | P032 | Lung | Upper lobe | pT2 | Female | 83 |  |
|  | P033 | Lung | Lower lobe | pT3 | Female | 64 |  |
|  | P034 | Lung | Upper lobe | pT2b | Male | 63 |  |
| Normal | P018 | Lung | Upper lobe | / | Female | 58 |  |
|  | P019 | Lung | Upper lobe | / | Male | 81 |  |
|  | P027 | Lung | Upper lobe | / | Male | 49 |  |
|  | P028 | Lung | Upper lobe | / | Male | 77 |  |
|  | P029 | Lung | Middle lobe | / | Female | 59 |  |
|  | P030 | Lung | Lower lobe | / | Male | 63 |  |
|  | P031 | Lung | Lower lobe | / | Male | 77 |  |
|  | P032 | Lung | Upper lobe | / | Female | 83 |  |
|  | P033 | Lung | Lower lobe | / | Female | 64 |  |
|  | P034 | Lung | Upper lobe | / | Male | 63 |  |

Supplementary Table 1. Clinical and pathological characteristics of lung tissue samples included in the single-cell RNA sequencing analysis.
